# The GLAMA (Girls! Lead! Achieve! Mentor! Activate!) physical activity and peer leadership intervention pilot project: A process evaluation using the RE-AIM framework

**DOI:** 10.1186/1471-2458-12-55

**Published:** 2012-01-19

**Authors:** Kate A Jenkinson, Geraldine Naughton, Amanda C Benson

**Affiliations:** 1 School of Medical Sciences, Discipline of Exercise Sciences, RMIT University, Melbourne, Australia; 2 Centre of Physical Activity Across the Lifespan, School of Exercise Science, Australian Catholic University, Melbourne, Australia

## Abstract

**Background:**

Implementing new initiatives and physical activity interventions in schools represents a myriad of challenges that if overcome can potentially facilitate a range of behavioural changes. The aim of this paper is to describe the process evaluation of specific design constructs used in the GLAMA (Girls! Lead! Achieve! Mentor! Activate!) peer leadership and physical activity pilot project. Conducted in a state secondary school in Australia, the intervention was designed to provide students with opportunities to develop leadership skills, school and social connectedness in addition to a range of physical activity experiences.

**Methods:**

This process evaluation used the RE-AIM (Reach, Efficacy, Adoption, Implementation and Maintenance) health promotion evaluation framework to assess three design constructs of the intervention: the effectiveness of leadership training and leader preparedness, activity suitability and participation, and the barriers to implementation of the intervention and potential solutions to overcome these barriers. As it was not the specific aim of this pilot, no behavioural change data were collected from students. Data were collected using a mixed methods approach including student questionnaires, teachers and researchers reporting on their own observations and feedback from students.

**Results:**

There were three main considerations evident across more than one RE-AIM dimension that need to be addressed to assist with future GLAMA dissemination. Firstly, the development of teacher, school and student participation. This needs to be through a variety of professional development opportunities for teachers, integration of the program within timetabled classes within the school and promoting the program to students as an opportunity to develop a range of skills to apply to future learning and workplace environments. Secondly, the successful translation of leadership training to practice is necessary to ensure that leaders are effectively able to motivate, facilitate and activate their teams. Finally, the need for consistent activity implementation requires sequential, competitive elements, purposeful team selection and clearly defined scoring and time periods for team 'challenges'.

**Conclusions:**

Factors that have the greatest impact on intervention success are those that come from within the school setting including: the structure of the curriculum, pressure to meet curriculum and assessment content, lack of support for new initiatives, multiple programs already running within the school, time allowances for teachers, appropriate training for teachers, and support for students to participate. These barriers need to be considered when developing all secondary school interventions.

## Background

Schools are recognised as key health settings and their importance in promoting knowledge of physical activity and healthy lifestyle behaviours via physical education and physical activity programs is well documented [1,2]. Despite this recognition, there are a lack of effective intervention strategies to promote physical activity in school children; therefore the development of effective physical activity interventions in schools continues to be a priority [3]. Nevertheless, promoting physical activity and healthy lifestyle behaviours among children and adolescents is a complex challenge [4], especially in a school context with many competing educational outcomes and institutional constraints.

School-based interventions are appropriate in many ways due to the level of continuous, intensive contact with students during their developmental years [5]. However, previously reported difficulties with implementing a range of interventions in schools have included the; lack of teacher participation, lack of program readiness, absence of program advocates, inadequacy of funding, reduction in infrastructure, poor association between the program's key features and organization routines, limited teacher training and support, insufficient amount of program materials, and inconsistent staffing [6-8]. Ultimately, effective interventions require the combination of careful planning and the engagement of the whole school community.

Notwithstanding the difficulties associated with implementing school-based interventions, the constant drive for schools and teachers to meet students' needs necessitates the adaptation of existing content as well as the successful implementation of new initiatives and interventions. Teachers are aware of their own difficulties facilitating engaging programs, especially in the area of physical activity and physical education [9]. Subsequently, teachers need to consider a range of teaching strategies, styles and methods for student engagement to ensure learning outcomes for all students.

One such strategy which involves peer assisted learning, encourages development across all learning domains. Peer assisted learning, teaching, tutoring or mentoring [10] are frequently interchanged terms. The commonality is that each strategy is underpinned by a learning process whereby students learn from and with others; this can be with students of the same-age or from those who are older (cross-age). Peer assisted learning in physical education and physical activity may overcome some aspects that impede student learning, enjoyment and participation by providing opportunities for increased levels of feedback, social learning and less direct instruction from the teacher [11]. This is particularly important for all adolescents, but especially girls who experience greater age-related declines in physical activity levels [12] and may not be attracted to the sometimes competitive, rigorous and the potentially uncomfortable nature of physical education [13].

Peer assisted learning appears to be an excellent vehicle for participant improvements to health/nutrition outcomes [14,15], physical activity participation including increasing on task behaviours [16-19], skill development [20-24], and self efficacy [23,24]. More specifically, a recent study of peer assisted learning in a physical activity leaders (PAL) program which used resistance training in adolescent boys reported significant reductions of several physiological outcomes [25], supporting previous findings from a lunchtime peer led activity program which also reported encouraging physiological changes in adolescents [26].

If peer assisted learning is conducted within a cross-age or same-age context then leadership opportunities are also provided for students. Whilst undertaking the role of 'peer tutor' or 'peer leader', the benefits reported have included; enhanced understanding of concepts, increased self determination, improved reorganization, clarification and knowledge building skills [27]. These leadership qualities are not exclusive to physical activity contexts. Promising peer assisted learning programs in remedial settings and other curriculum areas [28-31] highlight that programs outside those which are traditionally teacher-led may be successful in influencing student behaviour.

The RE-AIM health promotion evaluation framework [32] has been used to evaluate the multi-faceted components of interventions. The framework has previously been used in studies in primary school physical activity interventions [4,5,33] and community sport contexts [34]. The benefits of using the RE-AIM health promotion evaluation framework [32] are that it enables complex settings based interventions, such as those in school settings, to be comprehensively evaluated.

In summary, considering the potential benefits for students associated with peer assisted learning such as leadership development, increases in psychosocial and physiological outcomes in addition to increasing physical activity participation, an intervention in schools that provides opportunities to develop these components and can also engage girls should be considered. In an attempt to address the afore-mentioned parameters: the GLAMA (Girls! Lead! Achieve! Mentor! Activate!) peer leadership and physical activity intervention was developed. This paper aims to describe the process evaluation of the GLAMA pilot project and specifically focus on the evaluation of the intervention constructs including the;

i) Effectiveness of leadership training and leader preparedness

ii) Activity suitability and participation

iii) Barriers to implementation and solutions to overcome these to enable successful application in a wider school population.

## Method

The RE-AIM health promotion evaluation framework [32] was used to evaluate the integral intervention components. Evaluating the pilot is crucial to ensure its future development and dissemination is successful. Therefore, the use of a framework at the setting level as well as the individual participant level will assist the development of interventions that are applicable to the unique nature of school environments. Specific aspects of the program evaluated are detailed in Table 1.

**Table 1:** RE-AIM health promotion evaluation framework dimensions and definitions relevant to the GLAMA intervention at both individual and setting levels

Dimension	**How ability to reach dimension was measured**.
**Reach**	Refers to the representativeness of the school and the individuals' willingness to participate in the study. Reasons for non-participation were included after being gathered from teachers and participating leaders.

**Efficacy/** **Effectiveness**	Considers the effectiveness of the intervention at influencing primary outcome changes as well as assessing whether positive or negative outcomes were experienced by individuals or within the school setting.

**Adoption**	Refers to the schools acceptance of the intervention within the organization and examination of the factors that influenced that decision.

**Implementation**	Refers to the extent to which the participating students and school completed and made use of the various components of the intervention. This was measured by the level to which the main intervention components, including leadership training, activities and evaluations were completed as intended.

**Maintenance**	Refers to the extent to which schools and leaders maintained or continued with the intervention. This was difficult to assess given it was a pilot project.

Adapted from Austin, Bell, Caperchione & Mummery (2011)

### Intervention development

The intervention was designed to develop and foster leadership skills in Year 10 girls (peer leaders) so they were capable of leading a group of four to six girls of a younger age (Year 7) in a range of physical, cognitive and team focused activities. Based primarily on Social Cognitive Theory [35], the concept was also driven by the previous teaching experiences of the research team and our research into teacher perceptions, barriers and ability to implement physical education and physical activity in schools [9,36]. Ethical approval was obtained from both a University Human Research Ethics Committee and the State Department of Education and Early Childhood Development. Parental and participant dual consent was obtained for participation in the leadership program and for questionnaire completion.

**Figure 1:**
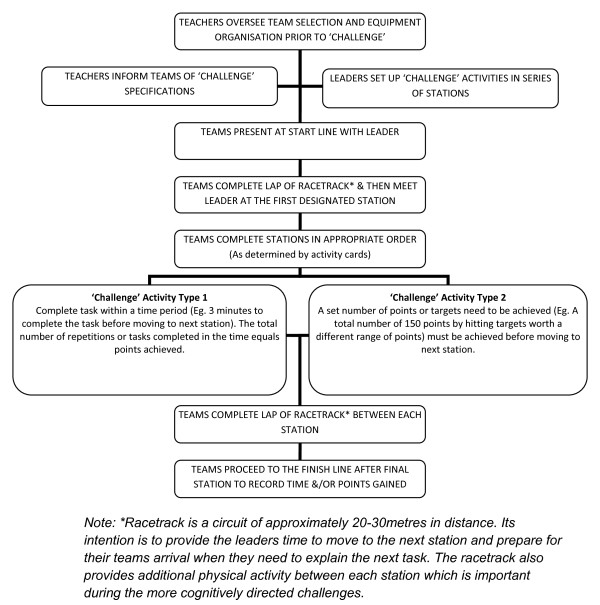
**Activity 'challenge' format**.

The activities used in the program were guided by an 'Adventure Racing' concept [37] and were based on providing opportunities to complete 'challenges' in groups before moving forward to the next activity. A 'racetrack' consisting of a lap of the gymnasium court was also included between activities. The basic structure of each 'challenge' is outlined in Figure 1. No 'challenges' required a high level of pre-existing motor skills or particular sporting attributes. Primarily, 'challenges' focused on team work, cognitive strategies, and opportunities to develop positive physical activity experiences. The venue for each 'challenge' was a school gymnasium, but activities could easily have been conducted in a range of indoor or outdoor environments.

### Participants

Participation by the state secondary school occurred after teachers indicated interest in a leadership and physical activity program. The rural school had a Student Family Occupation (SFO) Index rating (as determined by the state education department) [38] of medium which was within the desired low-medium rating for the study. A total of 67% of state secondary schools within the state currently have this rating. Three physical education teachers were involved in facilitating the project; two taught the Year 7 girls and one teacher recruited and liaised with Year 10 peer leaders to assist with the leadership training and program implementation.

**Figure 2:**
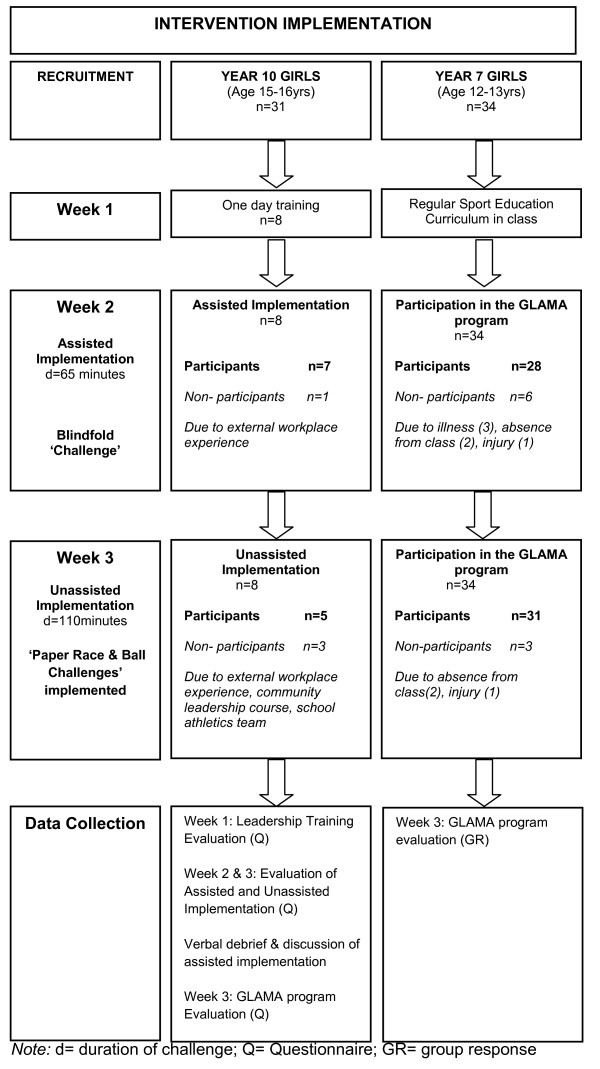
**GLAMA intervention pilot project implementation timeline**.

All Year 7 girls (12-13 years old) and Year 10 girls (15-16 years old) at the school were invited to participate via an assembly at which information was provided (Figure 2). Year 10 peer leaders were provided with music vouchers in appreciation of the time commitment required to lead the Year 7 students. Girls were chosen as our target demographic as they are often underserved in terms of encouragement and opportunities to partake in both physical activity and leadership development, particularly in rural communities [39]. Importantly, the declining participation rates, predominately in girls as they progress through secondary school were also considered a vital element to consider and attempt to address [12,13].

### Implementation

The intervention was conducted during October, 2010. Following recruitment, training was completed with the Year 10 peer leaders (Figure 2). We used a 'train the trainer' model which has been successfully incorporated in many health contexts [40,41] and appears to be appropriate in meeting outcomes in educational contexts [42,43]. The aim of the one day of leadership training conducted by researchers was to develop appropriate skills to enable peer leaders to lead their small team. The training specifically involved theoretical components, questioning, partner activities, brainstorming and group discussion to facilitate development. The following five key areas were addressed: understanding and developing leadership characteristics, developing communication skills, developing management skills to lead their group, and behaviour and motivation modification techniques. They also participated in the 'challenge' activities in which the role-modelling of both leader and participants took place.

The GLAMA program was conducted during the regular sport education [44] curriculum time. In the intervention school, the sport education program focused on gaining knowledge of game structures and strategies through participation in traditional games with few modifications. This is in contrast to fundamental skill development through activities and modified games provided in physical education. Therefore, teachers felt it was appropriate to implement 'team-based' activities in their curriculum with the GLAMA intervention able to meet similar outcomes (physical activity, team building, and social outcomes) to those encouraged in a traditional sport education unit.

The first assisted implementation was guided by the researchers and the three physical education teachers during week two. Year 7 teams were carefully chosen and considered friendship groups and positive, constructive relationships avoiding any confrontational issues which had concerned teachers previously. Peer leaders then completed the introduction session of 10 min with their Year 7 team including getting to know you activities and team identity formation through the establishment of rules and a team name. Teams and peer leaders then commenced the 'Blindfold Challenge'. The session was shorter than anticipated and went for 65 min due to externally imposed constraints of the school timetable.

The final unassisted implementation of the 'Paper Race' and 'Ball Challenge' occurred during week three. Due to three peer leaders missing, some teams were merged with others to accommodate this. Peer leaders independently led and implemented activities with their group with assistance only provided by teachers and researchers when setting up equipment. The session ran for the duration of class time, in total 110 min.

### Data collection

Data collection focused firstly on the one day training program provided for peer leaders (Figure 2). Data were collected using project specific questionnaires to evaluate the effectiveness of the training program in providing the required skills and engaging peer leaders in their forthcoming role. Secondly, the overall GLAMA intervention was evaluated by peer leaders, teachers and researchers after completion of the intervention. It entailed the use of a mixed methods approach including questionnaires, reporting on observations and feedback from students. The researchers implemented the training and observed all sessions conducted by the peer leaders. Evidence derived in a mixed method approach can offer guidance on how to create conditions for successful adoption, implementation and maintenance of interventions [5]. As it was not the intention, no behaviour change data were collected from students.

## Results

### Results for the dimension REACH

Two Year 7 girls' only classes with 34 girls in total were available for recruitment (Figure 2). The physical education teachers encouraged all girls to participate, however if they chose not to, they were offered alternative sport education opportunities in other classes during the intervention period. Participation during assisted implementation (n = 28) and unassisted implementation (n = 31) was high due to the program being run during curriculum time (Figure 2).

A total of 31 Year 10 girls were available for leadership training: eight girls completed the training (Figure 2). Year 10 peer leaders were difficult to reach with problems recruiting indentified as: existing commitments to a large range of school programs, reluctance to leave classes (as they would have to catch up on missed content), and unwillingness to work with Year 7 girls. There was also hesitation to engage in physical activity promotion or participation themselves despite the potential leadership advantages being gained and a small voucher being offered as an appreciation of their time and involvement.

The three teachers were accessible and responded to requests for information promptly. Both Year 7 physical education teachers agreed that they had difficulty throughout the year engaging their Year 7 girls in sport education and were appreciative of the opportunity to try a different approach in an attempt to engage their students.

### Results for the dimension EFFICACY/EFFECTIVENESS

#### Outcome 1: The effectiveness of leadership training and leader preparedness

Training priorities were to equip peer leaders with skills to understand content, competently deliver it and engage their teams. All training was implemented by the researchers. The results for leadership training and leader preparedness are shown in Table 2. Despite only one day of training prior to the program commencing, leadership training was positively rated amongst the eight peer leaders with 100% of leaders reporting that after the completion of training they had the confidence to lead a group of Year 7 girls through the program (Table 2). All peer leaders were ready (25%), very ready (63%) or extremely ready (12%) to lead their groups after training. Uncertainty surrounding leadership preparedness after the first assisted implementation related to difficulties with activities (remembering them the week following training), and understanding the written instructions. In evaluating the program, a total of 100% of peer leaders believed that their training equipped them with the skills to lead their team throughout the whole intervention (Table 2).

**Table 2:** The effectiveness of leadership training and level of leader preparedness in Year 10 student leaders

When	Outcome Measured	Description	Result	Sample Comments
**After Leadership training** **(n = 8)** **Week 1**	Leadership training	Would you be confident in leading a group of 4-5 Year 7 girls in the activities? ‡	Yes = 100%	N/A N/A
		
		Would you be confident in leading your peers in the activities?‡	Yes = 100%	

	Leader preparedness	1. How 'READY' are you to lead your group of Year 7 girls?¥	Ready = 25% Very Ready = 63% Extremely Ready = 12%	N/A

**After assisted implementation** **(n = 7)** **Week 2**	Leader preparedness	2. Did you find anything difficult about: a) any activities‡	No = 86% Not sure = 14%	"A bit hazy on most, needed showing what the activities were"
		
		b) working with year 7 students‡	No = 100%	N/A
		
		c) understanding written instructions‡	No = 86% Not sure = 14%	"Some were a bit confusing".
		
		d) using equipment‡	No = 100%	N/A
		
		e) comprehending the challenge‡	No = 86% Not sure = 14%	N/A
		
		f) leading the group‡	No = 100%	N/A

	Leader preparedness	3. Did you feel confident leading your group in the activities today? ‡	Yes = 86% Not sure = 14%	"I was excited to be a leader for the Year 7's".

	Leader preparedness	4. How 'READY'are you to lead your group of Year 7 girls again next week? ¥	Ready = 28.5% Very Ready = 43% Extremely Ready = 28.5%	N/A

	Leader preparedness	5. How 'MOTIVATED' are you to work with your group again next week?¥	Fairly Motivated = 43% Very Motivated = 43% Extremely Motivated = 14%	N/A

**Debrief session between implementations** **(n = 7)**	Leader preparedness	A debrief session provided for leaders to discuss their first session and address any difficulties that they may have experienced in preparation for the next implementation. No structured questions were asked.	N/A	N/A

**After unassisted** **implementation** **Program evaluation** **(n = 7)** **Week 3**	Leadership training	6. Did your training equip you with the skills to lead your team?‡	Yes = 100%	"It was hard to getting the girls motivated..." "We didn't have enough time to complete the challenge" "At the beginning everyone was shy..." "I had difficulty engaging them"

	Leader preparedness	Were you adequately prepared each week to lead your group?‡	Yes = 71% Not sure = 29%	N/A

		Did you like leading and working as part of a team to achieve the 'challenges'?‡	Yes = 86% Not sure = 14%	N/A

*Note: *‡ = 3 point Likert scale = "Yes", "No", "Not sure"; ¥ = 5 point Likert scale = "Not at all", "Close", "Ready", "Very", "Extremely"; Assisted implementation = first session where leaders were given assistance as required from teachers; Unassisted implementation = second session when leaders worked independently to lead their groups

Researchers' observations of the positive outcomes of training and ability to implement training objectives during the intervention included the peer leaders: prompt setting up and organization across all three 'challenges', selection and use of appropriate equipment, use of learned motivational techniques to encourage their team, quick movement between 'challenges', giving assistance when required to prompt their team in cognitive activities, use of activity cards and score sheets appropriately when difficulties arose.

#### Outcome 2: Activity suitability and participation

##### i) Year 7 Girls

The first activity, the 'Blindfold Challenge' was selected as it requires significant team work, communication, trust and a whole team contribution. The feedback teachers received from Year 7 students regarding the GLAMA program have been presented in Table 3. They have been grouped into common themes and focus on activity or program components, team work, and peer leader relationships. In summary, the Year 7 girls enjoyed many elements of the activities. However, the 'racetrack' component of the 'challenges' was not viewed quite as favourably (Table 3). Working with peer leaders, with peers and in teams was well received. In relation to affective development opportunities; girls wanted to choose their own groups, but also commented that swapping groups to work with others may also be of benefit to helping them get to know people.

**Table 3:** Participant responses to selected questions from the program evaluation (Year 7 and Year 10) following the GLAMA pilot program

	What was the best part of the GLAMA program?	What was the worst part of the GLAMA program?
**YEAR 7 RESPONSES (n = 31)**		

Activity/Program	"All the activities were fun" "The first weeks' activities (referring to the Blindfold Challenge)" "It was fun" "We liked that it was competitive" "Helps you to get fit"	"We had to run a lap after every activity" "The running a lap" "Running around the room"

Team Work	"Working in teams" "Working with others" "Working with my friends" "Working together" "Helps you to get to know people"	"We wanted to choose own group" "It was different because you weren't working with friends" "We should swap groups after every challenge"

Peer leader Relationships	"Working with a year 10 leader was good as they are not so cranky and are different to teachers" "Because the Year 10's are closer to age group...easier to connect to" "Lots of positive feedback from the leaders"	

**YEAR 10 RESPONSES (n = 7)**		

Activity/Program	"It was fun and exciting" "Everyone had fun" "It was mostly good" "It was great, except I missed the second session"	"That it was at the end of the day" "Packing up" "Filling out surveys"

Team Work	"Getting to work with and help the Year 7's" "It was something new...I got to work with people who I wouldn't normally" "Helping out the Year 7's"	"Some of the girls (Yr 7's) were a bit lazy to begin with"

Peer leader Relationships	"Being with the girls" "Communicating with the Year 7 girls and getting them motivated"	

In terms of active participation, researchers noted that Year 7 participation increased following the first implementation (n = 28) where three of six non-participants sat themselves out prior to commencing the activities. There was a different atmosphere during the second implementation (n = 31) when students were anticipating the next 'challenges'. Only one student was sitting out due to an injury, the other two students were absent from class. It was evident throughout three 'challenges' that students were engaged, working as a team and actively completed laps of the racetrack by either running or walking fast. Only one student decided not to take part in the final 'challenge' activity.

##### ii) Year 10 Peer Leaders

All seven peer leaders who completed the training and implemented at least one 'challenge' completed the program evaluation. One peer leader was only available for the training and did not implement any 'challenges'. As per Year 7, responses were themed (Table 3). To summarize the findings reported by peer leaders, the activities were perceived as fun. However, some aspects such as filling out surveys, the time of the day when delivered and packing up were small process issues which detracted from their enjoyment. Nevertheless, the experience of being peer leaders was underpinned by their enjoyment of being and communicating with, and helping the Year 7 students.

##### iii) Teachers

Both Year 7 physical education teachers were asked to respond to a series of questions relevant to the outcomes of peer leader preparedness, activity participation and suitability. Their responses have been compiled (Table 4) and highlight the suitability of the activities for motivating students to participate. The program also assisted in drawing attention to components of the activities that should be further considered; competitiveness, student groupings, timing of the activities in the school year, and engaging otherwise disengaged students.

**Table 4:** Physical education teacher responses to the GLAMA programs' ability to meet the primary outcomes

Teacher Responses	Leadership Preparedness	Activity Participation	Activity Suitability
Teacher (1)	"It was definitely beneficial having the year 10 students involved....younger students looked up to them and I think they almost wanted to prove themselves to them, to show them that they were capable of being mature and capable of performing skills. It was fantastic to see the Year 10's step up and take on a leadership role within the school." (1)	"During the first session we saw many of the students who do not usually participate having a go at all the activities (which was a big positive).....students worked well with students who they do not usually work with...enthusiasm and confidence increased. We saw some of the 'typical' non-participants pull out half way through activities during the second session. This could have been due to a decline in confidence as some of the activities were harder than the previous week's activities." (1)	"A program like this would be extremely beneficial for year 7 girls at the start of the year as many of the students would not have formed close friendships yet.....give students a chance to work with everyone in a positive team environment. In addition.......this allows students who are not so confident or do not like physical activity to participate in physical activity without even knowing it. Students such as this may see physical activity as a positive and fun thing rather than an exhausting task." (1)

Teacher (2)	"...Year 10 students seem to engage the Year 7 students. They were able to get students who don't normally participate to have a go, which was great to see." (2)	"Team work was one aspect that I think improved (through participation) as well as developing new friendships." (2)	"I would consider continuing on; it's just a matter of getting our hands on the right resources." (2)

### Results for the dimension ADOPTION

The physical education teacher who recruited the Year 10 peer leaders and completed the training with leaders was highly motivated in terms of implementing the GLAMA project. The two Year 7 physical education teachers were also supportive of the project. However, factors which hindered their full involvement included: a deficiency in knowledge of program development, program structures and implementation procedures. Other factors that may have influenced adoption were the schools extra curricula programs that occurred simultaneously and included another external leadership opportunity, sports team commitments, academic testing and workplace experience. Timetabling priorities also affected the first assisted implementation, with class time reduced to facilitate a presentation assembly.

### Results for the dimension IMPLEMENTATION

The pilot school implemented most components of the program. Factors limiting the implementation process included;

• Peer Leader availability: Seven peer leaders completed the first assisted implementation and only five leaders were available for the unassisted implementation. Absences were due to sporting team commitments, external school courses, workplace experience and other school programs (Figure 2).

• Year 7 participant absences: Absences were due to illness, sporting or other school commitments such as music lessons (Figure 2).

• Duration: Initially the pilot was designed over a 6 week period. This was to include 2 days of leadership training in the first week. This was to then be followed by four "in class" sessions for 65 min per week over a 4 week period (4 × 65 min). It would then conclude with the lunchtime sessions, one lunchtime session of 40 min per week for the duration of 2 weeks (2 × 40 min).

However, the school would only release the Year 10 leaders for 1 day of training. Additionally, it was decided between staff and researchers that the four single sessions could be provided in two double sessions, therefore including the exact same content but over a shorter duration (2 weeks rather than four weeks). Circumstances beyond the control of the researcher reduced one double session to only one single session.

The peer leaders were very reluctant to give up their time to commit to a program over a total of 6 weeks but were happy to do so over a lesser time frame. The peer leaders also considered their lunchtime as an important period of the day and therefore after discussions with them it was decided to remove the lunchtime component. Year 7 students when asked also suggested their own time to socialise at lunchtime was more important than participating in the program.

• Team selection: In this pilot program teams were selected by teachers prior to the program. However, one teacher noted that *"...perhaps, teamwork declined a little in the second session as some of the students believed they should have been given the opportunity to work with their friends..." (Teacher 1)*.

• Time and cost of intervention: All three 'challenges' involved equipment that was sourced from around the school and from the physical education resources already available. The main cost was in student time taken away from class. For the peer leaders, classes missed had to be made up in their own time. For Year 7 students, because it was during curriculum time the impact was minimal.

• Time of year for implementation: The program was implemented toward the end of the school year when friendship groups have already been established in Year 7. Year 10 peer leaders also had competing demands of preparing for exams.

### Results for the dimension MAINTENANCE

As this was a pilot intervention, maintenance was difficult to evaluate because of the short duration. However, a positive result was reported by physical education teachers at the intervention school during December 2010. Of the leadership group who completed training, seven of the eight Year 10 peer leaders continued their leadership at the school and were peer support leaders for the Year 7 students the following year.

A review of outcomes including the potential barriers and possible solutions to enable successful implementation and dissemination of this project in the future can be found in Table 5. The three main considerations that need to be addressed and were evident across more than one RE-AIM dimension include:

**Table 5:** Potential barriers and solutions for GLAMA intervention using the RE-AIM health promotion evaluation framework [32]

DIMENSION	POTENTIAL BARRIERS	POTENTIAL SOLUTIONS
**Reach**	***Future implementation of a school intervention of this design must consider the following to best target individual participants and school settings:***	

**S**	Implementation during school curriculum time.	Engage schools in program implementation during curriculum PE or Sport Education or potentially as an adjunct to 'Peer Support' Year 7 mentoring programs that many schools already provide. Students may not want to participate during their own free time such as lunchtime.

**S**	Have support and develop knowledge of the program with all teaching staff.	Develop support from school and staff by providing appropriate professional development and handout information prior to implementation. Must highlight benefits to staff and students of their own participation.

**S**	Ensure program is not competing against other school based programs for time.	Consult school calendar and highlight benefits of program for school transition, school connectedness, and psychosocial development.

**LT**	Recruitment of leaders may need a different approach.	Program needs to be promoted as an opportunity not a right; therefore incentives may not be needed. Leaders need to be aware of benefits. Link to community service programs such as Duke of Edinburgh is possible. There also needs to be consideration of recruiting leaders who are not already involved in similar opportunities and who sit outside the traditional 'leader' mould.

**LT**	Return of consent forms may be an issue.	If considered a 'compulsory' program by the school and fully supported, there may be a higher return rate of consent forms.

**Effectiveness** **/Efficacy**	***Consideration of the following will be needed to ensure leader competency, confidence and preparedness:***	

**LT**	Training protocol.	**OUTCOME 1: Leadership training and leader preparedness**. 1. Training programs should be clearly designed to meet appropriate outcomes to ensure that it will enable successful implementation of the interventions. Leaders should gain knowledge in the five key areas established in this pilot.

**LT**	Training duration.	An intervention that is implemented over a longer duration would require more training to be able to conduct more 'challenges' and greater understanding of group dynamics, leadership skills and how to problem solve. Refresher training just prior to the first implementation should be undertaken to help check for understanding and address any concerns or apprehensions.

**LT**	Length of time between sessions.	'Challenges' should be completed weekly to ensure a consistent team oriented approach otherwise leaders lose momentum and also understanding of tasks and their role.

**LT**	Reading and comprehending instructions for each activity.	Provide a booklet with all 'challenges' for leaders to take home and use to prepare. Ensure activity cards are clear and concise with diagrams and that leaders have opportunities to clarify before implementation.

**LT**	Opportunities to evaluate training and verbal feedback.	Leaders should be given the opportunity to provide both written and verbal feedback to help direct support they require to develop their leadership skills.

	***The following issues need to be addressed when providing activities for Year 7 students:***	

**A**	Sequencing activities correctly to engage students.	**OUTCOME 2: Activity suitability and participation** The first task completed should be challenging, engaging and provide an opportunity for students to contribute to team success.

**A**	Removal or adaptation of racetrack.	The racetrack element should be carefully considered in terms of its: length, application in more cognitively based activities to encourage activity, its benefits to leader organisation, its location and participant understanding of its purpose, how frequently it is used and the primary outcomes of the program challenge.

**A**	Adding competitive elements.	Scoring should be consistent between activity 'challenges', easy to use and fully explained in the activity cards and booklets leaders have.

**A**	Grouping of students in teams	Appropriate grouping of teams and also leaders to teams is paramount to intervention success and should be considered carefully. If leaders are working with other leaders, this should also be considered. Teams should be small, between 4 to 6 students if possible.

**LT**	Leader interest and understanding of activities and ability to motivate students.	Leader motivation and interest will be critical to Year 7 activity participation. All leaders should apply for positions of responsibility and potentially demonstrate they have the capacity to undertake this role. Leaders must complete training that promotes positive relationship building, communication skills, problem solving and ability to work with others in groups.

**A**	Disappointment in team/grouping.	Inappropriate grouping may lead to decreases in participation. Year 7 groups and students should be monitored throughout the program with groups confirmed as early as possible if changes are required.

**A**	Concern about being part of a losing team.	Bonus points can be given by supervising staff to leaders and teams for assisting with equipment, organization and appropriate 'team work' to reward desirable team related outcomes.

**A**	Time to complete the activity.	Time periods must be designated for each challenge and be consistent throughout the program. This will allow scoring to also be consistent between activities.

**Adoption**	***The following issues need to be addressed to promote setting adoption:***	

**S**	Teacher knowledge and support of the program.	See REACH 1.

**S**	School culture including previous lack of success with students, motivational issues with students and negative experiences with physical activity.	The program should be promoted to all students, with leaders comprehending the importance of the role they will play. Motivational issues and negative experiences with physical activity can be negated by limiting racetrack lengths, careful team selection, ensure leaders are motivating and encouraging and appropriate challenge activity selection to meet student needs.

**S**	Too many extra curricula activities already offered by the school.	1. See REACH 3.

**Implementation**	***To encourage successful implementation of this intervention, the following components need to be considered:***	

**S**	Leader availability for each session.	A consistent time every week needs to be provided for both leaders and students to ensure they attend, can plan for and contribute to each session. If leaders are absent, it impacts greatly on their peers and also other teams.

**S**	Participant contribution to team each session.	All participants should be held accountable for their team success after each challenge. This could be in the form of contributing individual points or overall team points. Teams crossing the finish line together and presenting to leaders together is also an important component in achieving this.

**S**	Consent.	See REACH 4.

**S**	Length of program and training within the school program.	The training duration provided for leaders has to equate to the period of implementation.

**S**	Team Selection.	See EFFECTIVENESS Outcome 2, 4.

**S**	Time and cost.	The outlay for equipment is minimal. The time taken for leaders to leave their classes to conduct the program is the most costly aspect of the program. Potentially timetabling a Year 10 and Year 7 class together for PE, Sport Education or Peer Support may alleviate this. Otherwise, classroom teachers need to be informed of when leaders will be missing and provide appropriate avenues for them to make up class time.

**S**	The time of year to deliver program the program to Year 7 students (weather, transition, exams, sport).	One of the aims of the program is to assist with Year 7 transition and therefore the optimum time for delivery is Term 1 or Term 2 of the school year. Optimal training time for leaders also needs to be taken into consideration, with exams and other commitments sometimes filling senior students' diaries. Weather will also impact on location/facility requirements.

**Maintenance**	***For a school to maintain a program and embed it within the school, the following parameters should be addressed:***	

**LT**	The duration of training and when to deliver the training.	A comprehensive training program should be undertaken to ensure leaders are competent and capable in leading their Year 7 teams. Provision of training periods should be included within the school day. The timing of training should also be considered otherwise refresher training will need to be provided. If the program is to be delivered at the start of a year, consideration needs to be given to leader selection and training beginning at the end of the previous year (see also EFFECTIVENESS Outcome 1, 2).

**S**	Impact on school having both year 7 and year 10 students participating in program.	All Year 7 students should have opportunities to partake in the 'challenges'. The biggest impact will be on Year 10 students who will have to miss classes if classes are not timetabled concurrently.

**S**	Staff required.	Staff training is required for those staff that will be assisting Year 10 leaders when the program is actually running. This will enable them to provide valuable feedback while the student leaders implement the program. Recruitment of key staff that will help drive and oversee the intervention is crucial to its success. Ongoing training of new staff to a school setting is necessary.

*Potential barriers include those relevant to: S *staff and school, *LT *Leadership training, *A *Activity/program design, selection or participation

i) Developing teacher, school and student participation.

ii) Translation of leadership training.

iii) Consistent activity implementation.

## Discussion

Overall engagement of the school, teachers and students was appropriate during the pilot. All teachers attended each session, there was an increase in Year 7 participation over the three 'challenges' and the lowest attendance by peer leaders was five of the available seven students during week three which was affected by external school activities.

Over a longer duration, gaining teacher and administration support in a school setting is imperative for intervention sustainability. Similarly to previous studies, we have found that having a 'program champion' to develop momentum and drive the program from within the school has shown to be influential in the success of school-based interventions [45,46]. Importantly, in conjunction with appropriate staff training [46,47], it can maximize opportunities for all involved and possibly enable the project to become embedded more broadly within the school culture.

For this peer assisted intervention to be successful it must be provided within timetabled lessons, and possibly collaborate with other programs with similar objectives (promote school and social connectedness, foster leadership, increase physical activity). Secondary school intervention studies such as Trial of Activity for Adolescent Girls (TAAG) [48] and Fitness Improvement Lifestyle Awareness (FILA) [26] have similarly found that the compulsory context of curriculum-based sessions are important in enabling greater opportunities for intervention success. It was evident in both our study and FILA [26] which involved peer assisted learning, that competing interests at lunchtime may impact on participation.

Supporting the developmental concepts of this pilot intervention, a recent systematic review of interventions that promote physical activity among young and adolescent girls' recommends that peer assisted learning strategies such as mentoring or tutoring should be one focus of future physical activity research [49]. Although the aim of the pilot was to specifically elucidate the effectiveness of the training for Year 10 leaders, the activities used with the Year 7 students and to see if there were any immediate barriers to the program within schools, it would be remiss to not address the fact that previous research has demonstrated the capacity to measure a range of different outcomes in peer learning contexts and these will need to be considered in the future implementation of the GLAMA program over a longer timeframe. These measures include physiological [20-24] as well as psychosocial outcomes [16-19,23,24]. Objective measures of physical activity for leaders and Year 7 students would be highly beneficial and relevant to the future implementation of the intervention.

One of the limiting factors of many peer assisted learning interventions is the integrity of the leader training protocol and whether the training provided to leaders is sufficient to secure the desired outcomes of the program [27]. We have found our training to be somewhat successful after assessing researcher and teacher observations as well as peer leader evaluations, although completing the implementation of three 'challenges' is not a true reflection of training success. Importantly, gathering support from students to complete the training and engage in the intervention also needs further consideration. Physical activity, peer assisted learning, or leadership opportunities are not attractive to all teachers or students. Further strategies would need to be considered on how to address these perceptions. Research has suggested that incorporating peer leaders to deliver interventions may possibly reduce the burden on teachers and may also promote responsibility in peer leaders and a greater understanding of the program resulting in higher retention [25]. These outcomes may be important for program champions to disseminate.

The design of the activities must provide opportunities for development across each of the learning domains: affective, cognitive and psychomotor. Supporting previous findings from the HIKCUPS study [50], our research has shown that detailed activities, equipment and the time for activities should be clearly available in manuals to clarify any difficulties peer leaders may have. These manuals should be provided for leaders in the future. Furthermore, recommendations from HIKCUPS also highlight that the selection of activities need to be engaging as well as health promoting [50]. Our findings concur, activities need to be carefully sequenced, provide competitive elements which participants reported they enjoyed, involve careful team selection and have clearly defined, consistent scoring, and time periods during individual sessions as well as the entire program.

## Limitations

Process evaluations are important components of intervention research [48]. The RE-AIM health promotion evaluation framework was used in this evaluation and has identified a range of different outcomes and limitations that should be considered prior to further implementation and dissemination of the GLAMA program. Firstly, the school chosen was rural, and despite being similarly ranked with two thirds of other schools within the Victorian state secondary system, the influences on rural students' participation in such programs may be different to their metropolitan counterparts. The sample of eight female leaders who undertook training and seven female leaders who implemented the project also limits external validity.

The use of self report lends itself to reliability issues, memory bias and problems with concentration and comprehension [4]. In an attempt to overcome these problems, questionnaires were completed immediately after training, program implementation and program completion. Timetable restrictions dictated timeframes for completion of tasks and may have influenced results; however, this is the reality of conducting interventions in school settings.

The duration of the intervention for 3 weeks does not provide knowledge of the long term difficulties within the setting and with participants to be fully understood. Although not the primary objective of this process evaluation, it also did not enable us to assess any immediate behavioural change in peer leaders or Year 7 girls, which is something interventions of a longer duration would need to consider.

## Conclusions

Despite barriers experienced by students and teachers at an individual level, the factors having the greatest impact on intervention success are those coming from within the school setting; the structure of the curriculum, timetabling, pressure to meet curriculum and assessment content, lack of support for new initiatives, multiple programs already running within the school, time allowances for teachers, appropriate training for teachers, and support of students to participate. A school's ability to adopt, implement and maintain programs needs to be considered most prominently in planning future implementation of school-based physical activity interventions [5,51] as well as those within other curriculum areas.

The GLAMA pilot intervention provided opportunities for leadership development, physical activity and social interactions for participants, all of which can be measured in its future application. Overall, it was a positive experience for Year 10 leaders, Year 7 girls and physical education teachers. The intervention should be revised using the recommendations from this study to further encourage a range of other school settings to adopt such programs, and considerations should include promoting involvement to both boys and girls in a cross-age environment over a longer duration.

## Competing interests

The authors declare that they have no competing interests.

## Authors' contributions

KJ contributed to the conception, design and implementation of intervention methodology, acquisition of data, analysis and interpretation of data, drafting, critical review and final submission of manuscript. AB contributed to the design of intervention methodology, acquisition of data, analysis and interpretation of data, drafting, critical review and final submission of manuscript. GN contributed to the design of the methodology, critical review of manuscript and final submission of manuscript. All authors read and approved the final manuscript.

## Pre-publication history

The pre-publication history for this paper can be accessed here:

http://www.biomedcentral.com/1471-2458/12/55/prepub
